# In Vitro Effect of Replicated Porous Polymeric Nano-MicroStructured Biointerfaces Characteristics on Macrophages Behavior

**DOI:** 10.3390/nano11081913

**Published:** 2021-07-25

**Authors:** Luminita Nicoleta Dumitrescu, Madalina Icriverzi, Anca Bonciu, Anca Roșeanu, Antoniu Moldovan, Valentina Dinca

**Affiliations:** 1 National Institute for Lasers, Plasma, and Radiation Physics, 409 Atomiştilor Street, 077125 Magurele, Romania; nicoleta.dumitrescu@inflpr.ro (L.N.D.); anca.bonciu@inflpr.ro (A.B.); antoniu.moldovan@inflpr.ro (A.M.); 2 Institute of Biochemistry of the Romanian Academy, 060031 Bucharest, Romania; radu_mada@yahoo.co.uk; 3 FOTOPLASMAT Center, 409 Atomiştilor Street, 077125 Magurele, Romania; 4 Faculty of Physics, University of Bucharest, 405 Atomistilor, 077125 Magurele, Romania

**Keywords:** nano and microstructured biointerfaces, polyvinylidene fluoride (PVDF), macrophage interaction, replication method

## Abstract

In the last decades, optimizing implant properties in terms of materials and biointerface characteristics represents one of the main quests in biomedical research. Modifying and engineering polyvinylidene fluoride (PVDF) as scaffolds becomes more and more attractive to multiples areas of bio-applications (e.g., bone or cochlear implants). Nevertheless, the acceptance of an implant is affected by its inflammatory potency caused by surface-induced modification. Therefore, in this work, three types of nano-micro squared wells like PVDF structures (i.e., reversed pyramidal shape with depths from 0.8 to 2.5 microns) were obtained by replication, and the influence of their characteristics on the inflammatory response of human macrophages was investigated in vitro. FTIR and X-ray photoelectron spectroscopy analysis confirmed the maintaining chemical structures of the replicated surfaces, while the topographical surface characteristics were evaluated by AFM and SEM analysis. Contact angle and surface energy analysis indicated a modification from superhydrophobicity of casted materials to moderate hydrophobicity based on the structure’s depth change. The effects induced by PVDF casted and micron-sized reversed pyramidal replicas on macrophages behavior were evaluated in normal and inflammatory conditions (lipopolysaccharide treatment) using colorimetric, microscopy, and ELISA methods. Our results demonstrate that the depth of the microstructured surface affects the activity of macrophages and that the modification of topography could influence both the hydrophobicity of the surface and the inflammatory response.

## 1. Introduction

There is an increasing interest in developing new and more performant biomaterials used as an implant for tissue engineering and drug delivery field. Nowadays, the optimization of implant surfaces relies on using synergetic effects of bulk materials, as well as biointerface characteristics, in terms of chemistry and topography, together with cellular responses. Besides its biocompatibility, thermal stability, chemical-mechanical properties, and piezoelectricity mimicking the extracellular matrix, PVDF was shown to have a carbon backbone molecular structure chemistry closer to biological tissues than that of ceramics [1]. All these could impact its use in tissue engineering applications, especially those related to bone or cochlear implants. PVDF was successfully used as suture material or surgical mesh, but in the last years, studies regarding tissue engineering applications of PVDF surfaces with different designs were explored, especially on bone, muscle, and neuronal cells. One of the main issues related to its biomedical application is related to hydrophobicity character, which can impair an efficient attachment of cells. Several physical and chemical methods have been used to improve PDVF surface functionalization and modifications, from blending with nanoparticles to grafting with hydrophilic hydroxyethyl acrylate (HEA) monomer via a radiation grafting method [2,3,4].

For example, scaffold-based PVDF such as PVDF-graphene oxide-polyvinyl alcohol, PVDF-graphene oxide, and PVDF-collagen-platelet-rich plasma nanofibers were reported to induce a high osteogenesis rate of human-induced pluripotent stem cells in vitro, thus being promising candidates for bone tissue regeneration [5,6,7]. PVDF scaffold obtained by electrospinning has been reported to promote the attachment and osteogenic differentiation of human mesenchymal stem cells [8]. Tuning surface topography and creating biomimetic modified PVDF films led to increased MG-63 human osteoblast-like cells adhesion and collagen formation compared to smooth surfaces [9]. A novel PVDF substrate deposited by MAPLE and subsequent thermal treatment as thin hydrophilic coatings with different roughness was able to promote the adhesion and proliferation of MC3T3-E1 pre-osteoblasts [10]. PVDF film on a Ti substrate conducted to induction of osteogenic differentiation in bone marrow mesenchymal stem cells (BMSCs), especially on polarized surfaces [11], the same behavior being observed for human adipose stem cells (hASCs) on poled β-PVDF films [12,13].

It is known that inflammatory response is the first important reaction of the body to material implantation [14]. Among the first immune cells interacting with material surfaces are macrophages, key cells in the modulation of the host inflammatory responses [15]. Therefore, understanding how the characteristics of biomaterial surfaces can influence or trigger macrophages behavior is crucial. Regarding micro or nanostructured PVDF, a limited number of studies are related to the effects on cells—especially macrophages—involved in the host response to the foreign body [16,17]. Within this context and given the potential of PVDF to be used as stable and multifunctional material in various clinical applications, physical-chemical characteristics of porous replicated PVDF biointerfaces along with biological in vitro capacity to induce the immunomodulation of macrophages were analyzed in our study.

## 2. Materials and Methods

### 2.1. Materials and Reagents

Poly (vinylidene fluoride)-(PVDF beads, Mw~180,000 by GPC); N,N-Di (methylformamide) (DMF solvent, ACS reagent, ≥99.8%) and the (Hydroxypropyl) methyl cellulose-(HPMC, viscosity 40–60 cP, 2% in H_2_O (20 °C) (lit.)) were purchased from Sigma-Aldrich (Merck, Saint Louis, MO, USA) and used without further purification. Polydimethylsiloxane (PDMS; Sylgard 184 Silicone Elastomer Kit) was obtained from Dow Corning (Midland, MI, USA).

### 2.2. Method

#### 2.2.1. Pyramid-Shaped Micropatterns PDMS Mold

Initial molds based on polycarbonate (PC) multi-scaled inverted pyramidal laser micromachined surfaces (with lateral dimensions of 8 microns and three depths 1.5; 3, respectively 5 microns) obtained as described in [18,19] were used for obtaining the PDMS secondary pyramidal structures molds. The PDMS (10 to 1 mix ratio elastomer and initiator) was placed onto the PC mold for 48 h at room temperature under the fume hood. The resulting PDMS samples were subsequently removed from the mold and cleaned twice by ultrasonication, each 10 min cleaning step being performed in Millipore ultrapure water (Supplementary Figure S1).

#### 2.2.2. Porous Microstructured PVDF Preparation

For the preparation of the solution, the PVDF beads were blended in DMF solvent at 20 wt% polymer/solvent final ratio, vigorously stirred at 500 rpm using a magnetic stirring bar (Thermo Fisher Scientific, Waltham, MA, USA) at 80 °C constant plate temperature until a transparent and homogeneous solution was obtained. In order to ensure an appropriate detachment of PVDF material from the PDMS replica, 1 wt% HPMC (Sigma-Aldrich (Merck) KGaA, Darmstadt, Germany) solution was spin-coated at 1000 rpm (Laurell, Laurell Technologies Corporation, North Wales, PA, USA) on the PDMS patterned pyramid-like/shaped structures surface.

The PVDF solution was poured on the HPMC coated PDMS molds and placed under the fume hood for 68 h at room temperature (RT) following other for 4 h at 60 °C on a hot plate. In this way, the complete evaporation of the DMF solvent from the final replica PVDF along with the annealing process inducing β-PVDF formation were achieved [20]. The replicas were then demolded and washed in distilled water to eliminate any trace of residual HPMC.

The final dimensions of the Replicated PVDF reversed pyramidal microarrays are decreasing, with smaller values compared with those on the PDMS mold (lateral dimension of 8 microns) due to polymer shrinkage after thermal treatment and DMF solvent evaporation processes. The replicated structures will be referred to as P1, corresponding to 0.8 µm depth, P2 for 1.5 µm and P3 for 2.5 µm and with the lateral size of 4 microns. In the final Replicated PVDF samples shrinkage can be around to 50% and the exactly reversed pyramidal patterns dimensions are determined by the SEM and AFM measurements. Nevertheless, it is possible that the inherently high surface pore size of the PVDF polymer can superimpose the size of the final structure. Therefore, this particular factor must be considered, depending on the final application [21,22].

### 2.3. Microstructured PVDF Replica Topography Analysis by SEM and AFM

#### 2.3.1. Scanning Electron Microscopy

Topographical observation of the porous microstructured surfaces was investigated by Scanning Electron Microscopy(SEM) with a JSM-531 Inspect S, system (Hillsboro, OR, USA) at an accelerating voltage of 20 kV. The samples were air-dried and covered using a sputtering coater with 10 nm Au prior to microscopy investigations (Agar Scientific Ltd., Essex, UK).

#### 2.3.2. Atomic Force Microscopy

Atomic Force Microscopy (AFM) was used to determine the depth profiles created by the existing pyramid-type structures on the PDMS mold and to evaluate the morphological features the overall roughness values (rms) of the samples (several planes and 3D areas with dimensions 20 µm × 20 µm, 30 µm × 30 µm) by a Park XE 100 AFM system (Park Systems, Suwon, South Korea), in ambient conditions, with silicon tips, in non-contact mode.

### 2.4. Wettability and Surface Free Energy Characterization

#### 2.4.1. Contact Angle (CA)

The surface wettability of PVDF samples was measured by using the sessile drop method applied at constant temperature of 20 °C. A droplet (4 μL) was placed on the surface of samples and the contact angle measurements were performed using a KSV CAM101 microscope (KSV Instruments Ltd., Espoo, Finland) equipped with a video camera and FireWire interface, which allowed the acquisition of images with a resolution of 640 × 480 pixels.

#### 2.4.2. Surface Free Energy Measurements (SFE)

The surface free energy (SFE) was calculated using two wet agents: deionized water as a polar liquid, and for the completely dispersive liquid, di-iodomethane. The final SFE values were extracted from contact angle measurements and conducted using the concept of polar and dispersion components by means of the Wu’ calculation method/approximation [23,24].

### 2.5. Replica PVDF–Chemical Profile: FTIR and XPS Analysis

#### 2.5.1. Fourier-Transform Infrared Spectroscopy (FT-IR) Analysis

To evaluate the specific chemical profiles of the material, the chemical structure of the samples (pristine PVDF, replica PVDF) was investigated by Fourier-Transform Infrared Spectroscopy (JASCO INCORPORATED28600 Mary’s Court, Easton, MD 21601, USA) (FT-IR) analysis. The main characteristic IR vibrations of functional groups were compared to those of the pristine material using a Jasco FT/IR-6300 type spectrometer (JASCO Inc. 28600 Mary’s Court, Easton, MD 21601, U.S.A in the 400–7800 cm^−1^ range, with a resolution of 4 cm^−1^. All FT-IR spectra were measured in absorption mode by the accumulation of 1024 scans, while the pristine PVDF (pressed bead) was measured in ATR mode (Attenuated Total Reflectance).

#### 2.5.2. X-ray Photoelectron Spectroscopy

An Escalab Xi+ system, Thermo Scientific (Waltham, MA, USA), was used for X-ray photoelectron spectroscopy (XPS) survey and high-resolution XPS spectra acquisition. The survey scans were acquired using an Al Kα gun with a spot size of 500 µm, pass energy of 50.0 eV, and an energy step size of 1.00 eV (5 scans). Furthermore, the excessive charging of the samples was diminishing using a flood gun. For the high-resolution XPS spectra the pass energy was set to 10.0 eV, the energy step size was 0.10 eV; 15 scans were accumulated for O1s, 10 for C1s, and 5 for F1s.

### 2.6. Biological Investigations of PVDF Replicated Surfaces

#### 2.6.1. Sterilization Procedure

Before in vitro evaluation, all materials were sterilized for 30 min in antibiotics (penicillin10,000 Units/mL-streptomycin, 10,000 μg/mL, Gibco by Life Technologies, Thermo Fisher Scientific, Waltham, MA, USA) in 1% phosphate -buffered saline (PBS) solution for preventing microbial contamination.

#### 2.6.2. Cell Culture

THP-1 cells, human monocyte cells (ATCC, TIB-202) were maintained in RPMI 1640 medium with 1% (*v*/*v*) streptomycin/penicillin and supplemented with heat inactivated 10% fetal bovine serum (FBS, Gibco by Life Technologies, Paisley, UK) at 37 °C in a humidified atmosphere with 5% CO2 levels. Mycoplasma-free cells were routinely cultured at a density of 2 × 10^5^ cells/mL every 2 to 3 days. Cell viability was monitored using 0.4% trypan blue dye staining. Cells with more than 90% viability and up to 10 population doublings were used for this study. All experiments involving THP-1 cells were performed in triplicate, twice, n = 6.

#### 2.6.3. In Vitro Macrophage Stimulation Assay

THP-1 cells were seeded at 4 × 10^5^ cells on material surfaces in 24-well tissue culture plates (Costar, Corning Inc., Corning, NY, USA) and differentiated to macrophages by 48 h incubation with 10 ng/mL phorbol 12-myristate 13-acetate (PMA, Sigma-Aldrich, Saint Louis, MO, USA, P8139). At the end of 2 days, THP-1 cells were washed with incomplete medium and placed in glutamine-free RPMI 1640 medium supplemented with 5% (*v*/*v*) FBS (medium cell assay, MCA) for 4 h resting period before incubation with either 10 ng/mL lipopolysaccharide LPS (Escherichia coli 055: B5, L4524) from Sigma-Aldrich (Saint Louis, MO, USA) or MCA for a further 18 h to simulate pro-inflammatory and non-inflammatory experimental conditions (control).

#### 2.6.4. Cell Viability

Metabolic activity of THP-1 macrophage cells cultured on different topographic surfaces in the presence and absence of inflammatory stimulus was assessed using MTS ([3-(4,5-dimethylthiazol-2-yl)-5-(3-carboxymethoxyphenyl)-2-(4-sulfophenyl)-2H-tetrazolium, inner salt]) assay (CellTiter 96^®^ AQueous One Solution Cell Proliferation Assay, Promega, Fitchburg, WI, USA) according to the manufacturer’s instructions. The in vitro quantitative colorimetric method is based on conversion of tetrazolium compound to a formazan dye by mitochondrial dehydrogenases from viable THP-1 cells. Cells (4 × 10^5^/well) were seeded onto the material surface or coverslip and after 3 days of differentiation followed by 18 h incubation with LPS or medium. Then, cell medium was exchanged with a mix of MTS reagent and cell culture media, and after 0.5 h incubation in standard culture conditions, absorption at 450 nm was using a microplate reader (Mithras Berthold LB 940, Berthold Technologies, Bad Wildbad, Germany).

#### 2.6.5. Cell Adhesion and Morphology

4 × 10^5^ THP-1 cells were grown on each material surface in 24-well cell culture plates as described above. After 18 h of incubation with or without inflammatory stimulus at 37 °C, the culture medium was discarded and cells were washed with PBS. For cell adhesion and morphology evaluation on topographic surfaces, the specimens were prepared for Scanning Electron Microscopy (SEM) examination as reported previously [25] Briefly, cells were immediately fixed for 20 min at RT with 2.5% glutaraldehyde solution in PBS and dehydrated with a series of ethanol concentrations 70%, 90%, and 100%, two rounds of 15 min. for each concentration. Then the cells were subjected to hexamethyldisilazane (HDMS, Sigma-Aldrich, St. Louis, MO, USA) gradient drying using 50%, 75% and 100% in ethanol for two times per 3 min. for each solution. The samples were kept in a dry environment in a chemical Euroclone AURA 2000 M.A.C. fume hood before subsequent preparation procedures.

In order to quantitatively assess morphology changes of THP-macrophage on material surfaces, SEM images were analyzed using Image J Programme, Java 1.5.0_06 (Rasband, W.S., ImageJ, U. S. National Institutes of Health, Bethesda, MD, USA, https://imagej.nih.gov/ij/, 1997–2018). The samples were air-dried and covered using a sputtering coater with 10 nm Au prior to microscopy investigations (Agar Scientific Ltd., Essex, UK). Objects were configured for threshold, set for scale, measured using ROI Manager and cell area was calculated and expressed as μm^2^.

#### 2.6.6. Cytokine Secretion

Enzyme-linked immunosorbent assay (ELISA) was used for the detection of inflammatory cytokines secretion following in vitro macrophage stimulation with 50 ng/mL LPS for 18 h. After incubation with or without stimuli, the cell culture was collected and centrifuged at 3000 rpm for 5 min to remove cell debris. Supernatants were stored at −80 °C until cytokine measurement. The samples were thawed, diluted, and pro-inflammatory cytokine concentrations were assayed using specific DuoSet ELISA kits for TNF-α and IL-6 cytokines and DuoSet Ancillary Reagent Kit according to supplier’s recommendations (R&D System, Minneapolis, MN, USA). The optical densities were measured at 450 nm in an ELISA reader (Mithras LB 940 DLReady spectrophotometer (Berthold Technologies GmbH & Co. KG, Wildbad, Germany) Devices, Berkshire, UK). The cytokine concentration was expressed in pg/mL based on a specific standard (recombinant TNF- α and IL-6) curve for each cytokine.

#### 2.6.7. Statistical Analysis

All values are expressed as mean ± SD. Data were analyzed using Student’s *t* test and one way ANOVA, followed by Bonferroni post hoc testing using GraphPad Prism software for Windows version 5 (GraphPad Software, Inc., LaJolla, CA, USA). Statistical significance was set at *p* < 0.05.

## 3. Results and Discussions

### 3.1. PVDF-Replica Morphology Analysis

Various reports consider the influence of topographies, micro-features, internal architecture, roughness, and porosity of biomaterial surfaces as important factors for modulating the inflammatory response of macrophages [26].

**Figure 1:**
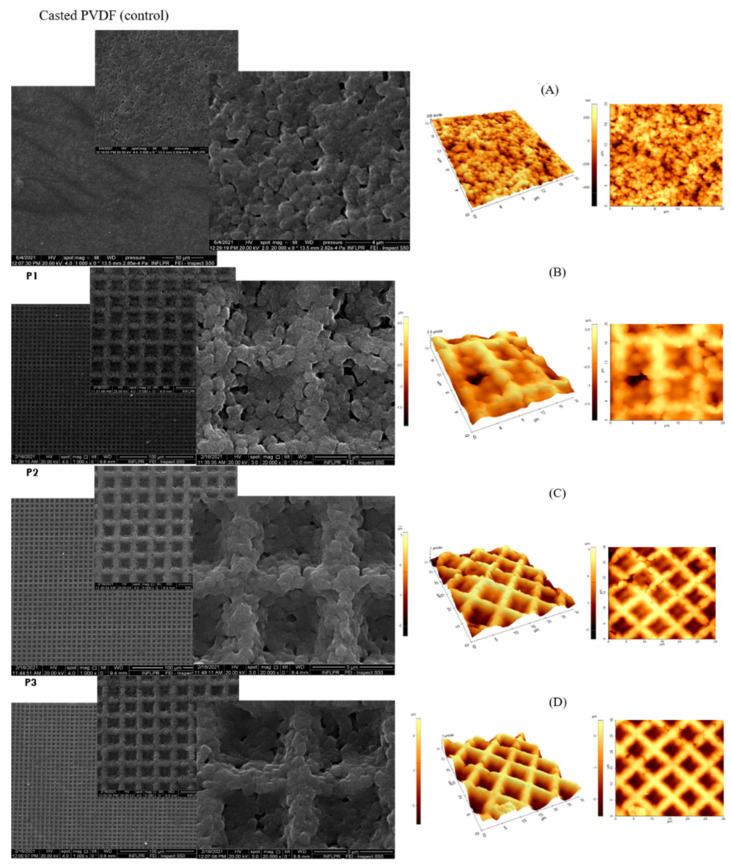
SEM micrographs (**left**) and AFM surface morphology images (**right**) of casted and replicated PVDF (30 × 30 μm^2^): casted PVDF (**A**) and microstructured PVDF replicas annealed at 60 °C with different depths: (**B**) P1: 0.8 μm, (**C**) P2:1.5 μm, (**D**) P3: 2.5 μm.

The changes in the surface topographies and morphologies of replicated PVDF surfaces compared to casted PVDF (control) are shown in Figure 1A–D. Both types of surfaces are characterized by a rough and porous aspect, with nano- and micro-sphere-like features (110 to 670 nanometers) which appear to be bound together to form a network linked together into a granular and porous blanket. SEM images revealed uniformly replicated squared wells with 4 microns lateral size (reversed pyramidal structures), maintaining the geometrical characteristics of the molds but with shrinkage of lateral dimensions of 50% (Figure 1B–D).

### 3.2. Chemical Characterization of the Replicated PVDF

**Figure 2:**
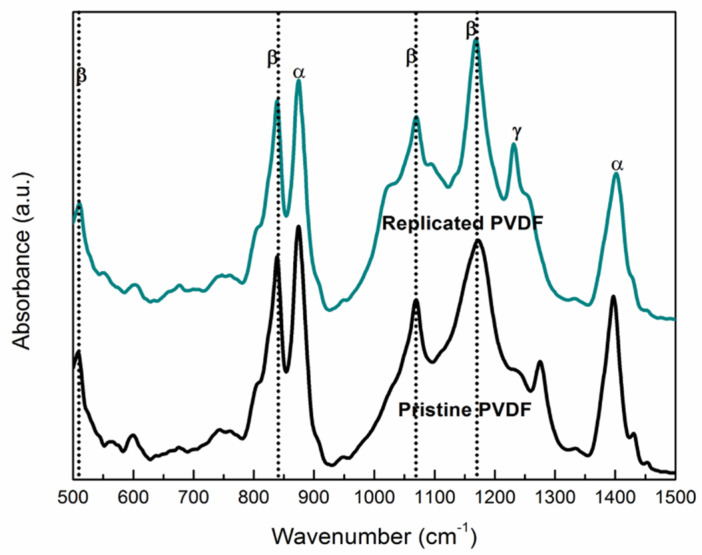
FT-IR spectra of Pristine PVDF (black line) and Replicated PVDF (colored line).

FT-IR analysis has been used to identify the chemical signature patterns of PVDF from the pristine material in comparison with the PVDF replicated samples (Figure 2). The FT-IR analysis of pristine PVDF (black line) confirms the existence of two phases, α- and β-phase. These chemical patterns originate from oscillations of large parts of the PVDF carbon backbone and/or attached specific/unique functional groups (C/F and O/C). Thus, the main peaks of IR vibration modes of Replicated PVDF (blue line) for the α-phase are around at 480, 763, 876, and 1402 cm^−1^, whereas for the β- phase the peaks are about 511, 603, 839, 1071, and 1170 cm^−1^ [20,27]. The peak at 1233 cm^−1^ is assigned to the γ- phase, while the peaks at 763 cm^−1^ belonging to α-phase are assigned to the CF2 bending (rocking vibration) [27,28]. Moreover, the peak found at 839 cm^−1^ was related to C–C–C asymmetrical stretching vibration and CF stretching vibration of PVDF respectively [29]. The spectrum retained the specific absorption peaks of the pristine PVDF in the final Replicated PVDF (Figure 2-blue line). In the case of the β- phase, the peak at 839 cm^−1^ is attributed to a mixed-mode of CH2 rocking and CF2 asymmetric stretching vibration which exists in both pristine and PVDF replica [20]. One can see that the peak at 1276 cm^−1^ has been shifted to 1233 cm^−1^ which is assigned to the γ- polymorph phase and correlated to pyroelectric property, see Table 1 [30,31]. Moreover, peaks in β-phase such as 839 cm^−1^ which corresponds to C–F stretching vibration and which are attributed to piezoelectric properties are enhanced [31,32,33] while other peaks in α-phase such as 763, 876, and 1402 cm^−1^ (CH2 wagging vibration) are diminished in the absorption spectrum of replicated PVDF [20]. However, there were no significant differences between pristine material and PVDF replica indicating that during the replication the material did not undergo chemical changes or more [29].

**Figure 3:**
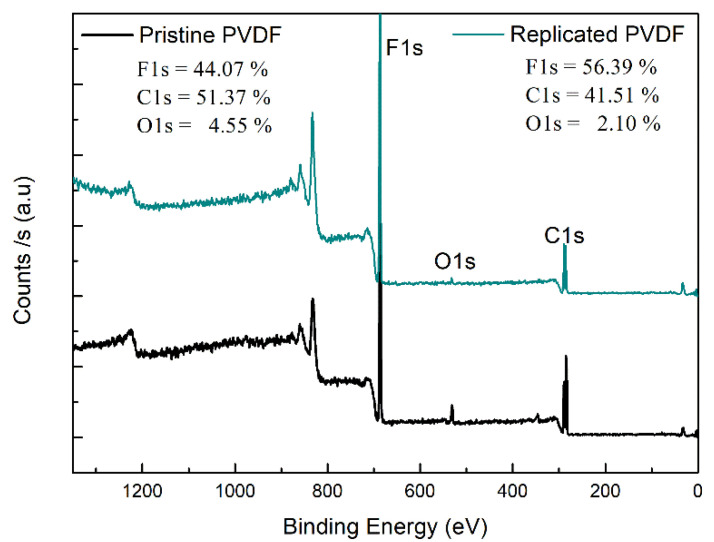
X-Ray photoelectron spectroscopy (XPS) wide survey spectra scan: Pristine PVDF (black line) and Replicated PVDF (colored line).

The surface physical and chemical characteristics of the sample is one of the key parameters that can influence the activation status of macrophages [34]. The chemical surface characteristics changes of the pristine and replicated PVDF material were determined by X-Ray photoelectron spectroscopy (XPS) analysis. Fluorine, oxygen, and carbon, elements were found in both samples as shown in the XPS wide survey spectra (Figure 3).

**Figure 4:**
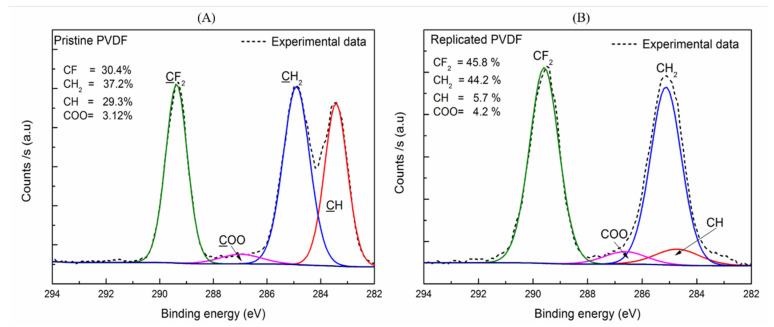
X-Ray photoelectron spectroscopy (XPS) high resolution spectra scan of C1s (carbon components): (**A**) Pristine PVDF and (**B**) Replicated PVDF (green CF2, blue CH2, red CH, pink COO).

For Pristine PVDF, strong peaks attributable to F1s, O1s, and C1s were found. Further, we found no significant differences result of the XPS measurements onto replicated PVDF. In addition, we analyzed the high-resolution spectra to identify the different groups, on the polymer surface, of C1s core-level, represented in Figure 3. To intuitively show if the inherent hydrophobic character of the Pristine PVDF scaffolds is suffering some changes after thermal annealing treatment (4 h at 60 °C), the carbon atomic percentage in total was quantitatively calculated according to the peak areas where the characteristics peaks are highlighted. In the Pristine PVDF the main peaks are centered at 289.4 eV (due to CH2-CF2-CH2), 284.8 eV (due to CF2-CH2-CF2), 283.4 eV (CH) [35,36,37] and for 286.98 eV for (COO) represented in Figure 4A. Similar results were obtained for Replicated PVDF (Figure 4B), 289.6, 285.1, 284.8, and 286.6 eV due to components CF2, CH2, CH, and COO, respectively.

These results indicate that the replication process maintains its hydrophobic nature of the surface characteristic since the F/C ratio content is slightly increased on the surface after replication. No de-fluoridation was observed where the replicated PVDF had nearly the same behavior as in the PVDF-pristine case. The atomic composition for the PVDF surfaces was obtained from the wide survey spectra (Figure 3). The fluorine-to-carbon atomic ratio (F/C) increases from 0.86 to 1.36 for the PVDF-replica. On the other hand, an increase occurs in the oxygen to carbon ratio was O/C from 0.08 to 0.46 after replication, showed in Table 2, where the values for the Pristine PVDF are similar to those found in the literature [38]. Therefore, it is entirely plausible to assume that the initial polymer reaction with the DMF solvent and the thermal treatment is not involved in breaking of the main bonds. Results of XPS analysis for the F/C and O/C atom ratios of the PVDF surfaces before and after replication are summarized in Table 2.

### 3.3. Wettability

Wettability (hydrophobicity/hydrophilicity), roughness, and porosity of biomaterial surfaces are characteristics that could modulate some processes such as cell attachment or growth, viability, and inflammatory response. In literature, studies demonstrated that hydrophilic surfaces could induce less inflammation while the hydrophobic surfaces are associated with the up-regulation of the pro-inflammatory response by macrophages [26,39,40].

**Figure 5:**
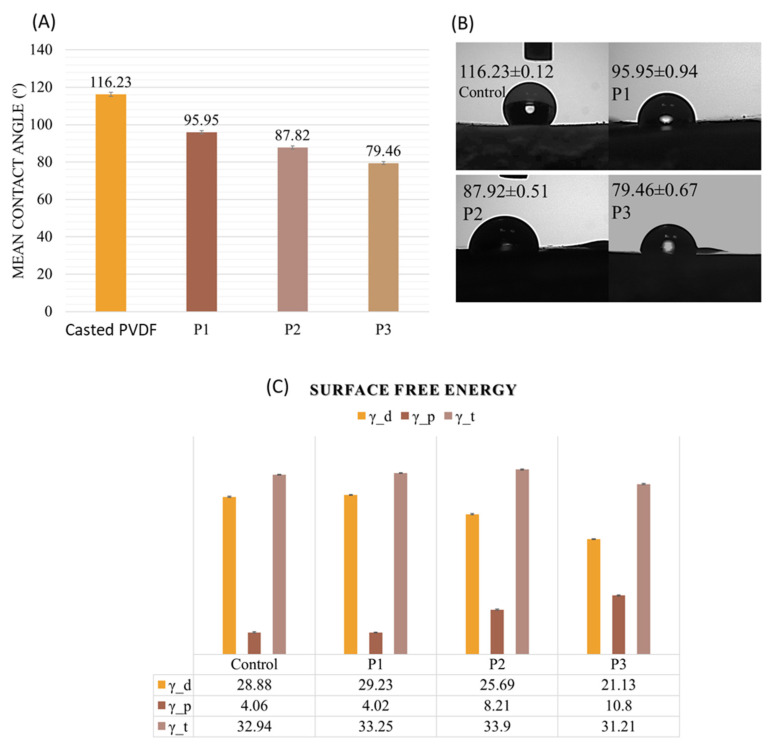
Histogram of contact angles (CA) of PVDF samples (**A**), optical images of the droplets onto surfaces and their corresponding CA (**B**), and the histogram of surface free energy (SFE) of casted and replicated PVDF (microstructured PVDF replicas annealed at 60 °C with different depths: P1 0.8 μm, P2 1.5 μm, P3 2.5 μm) (**C**).

Due to its fluorinated composition, the PVDF material naturally exhibits high water contact angle values of around 85° to 130° [40]. In our case, the casted PVDF samples were characterized by a contact angle value of 113°, which confirms its inherent hydrophobicity. However, a transition from hydrophobic to moderate hydrophilic values was observed in the case of nano-microstructured PVDF replicas (Figure 4A) From a contact angle value of 96° for the P1 structure to a contact angle value of 80° for the P3 sample (see Figure 5). These results may reflect in the final WCA values where we assume is the water droplet’s ability tends to penetrate the internal scaffold architecture (on the capillary action) [41].

The water contact angle (WCA) is directly related to the surface energy, and also dependent on surface morphology and chemical functional groups present on the surface. In its study Wenzel indicates that the water contact angle of the surface decreases with increasing surface roughness when the surface is composed of hydrophilic substances [41,42]. In our study, the root mean square (RMS) analysis revealed changes in surface roughness values from 0.3 µm for P1 to 0.6 µm for the P2 sample, 1 µm for the P3 sample, respectively, correlated to the decrease in wettability.

The results presented in Figure 5C show that the value of surface free energy for control PVDF is 32.94 mJ/m^2^, which is in good agreement with the Wu’s surface energy data by harmonic mean equation, γ_t = 33.2 mJ/m^2^ [23]. No significant total surface free energy differences between the casted and replicated samples were observed. The data indicate that the replicas are characterized by a preponderantly increase of the polar component of the surface energy (4.02 mJ/m^2^ to 10.8 mJ/m^2^). Harnett et al. [43] reported that a polar component higher than 5 mN/m led to increased cell spreading and this finding is highly suggestive that PVDF replicas obtained by us could be able to improve the material performance by favoring the tissue/material interaction at the targeted cellular level. Accordingly, to F. Gentile et al., cells preferentially grow on rough substrates, favoring cell adhesion [44].

### 3.4. Biological In Vitro Investigations

One important aspect in biomaterial design is to manipulate the physical-chemical surface properties in order to achieve an adequate host immune response. Since macrophages are the drivers of this response, much of the studies focused on the decrease of macrophages-associated inflammation to biomaterial.

Cell-material surface interactions were investigated using human premonocityc THP-1 cells differentiated to macrophages, the most widely used in vitro model for cell response evaluation to pro-inflammatory stimuli [45].

#### 3.4.1. Cell Viability

**Figure 6:**
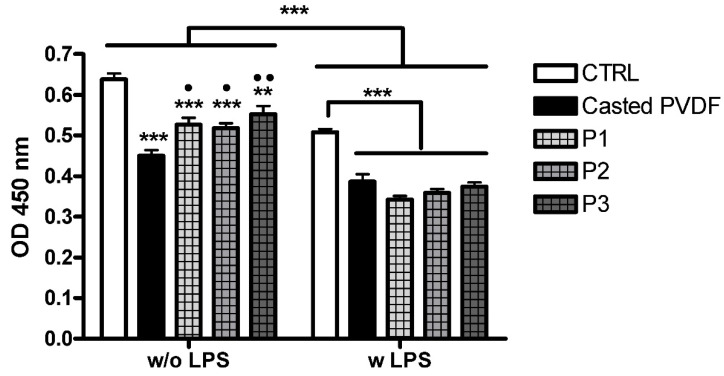
Effect of PVDF scaffolds on metabolic activity in LPS-activated and inactivated THP-1-differentiated macrophages cells. Cells cultured on glass coverslip and treated with cell assay medium served as negative controls (CTRL). Data are shown as mean ± standard deviation values of triplicates in each group. *** *p* < 0.001, ** *p* < 0.01 vs. CTRL, •• *p* < 0.01, • *p* < 0.05 vs. Casted surfaces.

The viability of THP-1-differentiated macrophage grown on different surfaces was evaluated using metabolic activity MTS assay. The results presented in Figure 6 showed that the viability of cells grown on inverted pyramidal surfaces was statistically higher compared to the casted polymer surface (*p* < 0.01 for P3 or *p* < 0.05 for P1 and P2), but lower than the control (coverslip), in the absence of inflammatory stimuli (*p* < 0.001 for P1 and P2, *p* < 0.01 for P3).

When LPS was added to activate macrophages, a statistically relevant decrease (*p* < 0.001) in the viability of cells grown on all surfaces compared to unstimulated cells was observed. In the case of inflammatory induced conditions, no significant changes in the cells’ viability were observed between the casted polymer and topographically modified scaffolds. However, cell viability was lower for all PVDF scaffolds than the CTRL (*p* < 0.001).

It has already been shown that the treatment of THP-1 differentiated macrophages with LPS can cause cytotoxic effects depending on time of exposure, concentration of inflammatory stimulus and PMA as well as biomaterial characteristics [25,46,47]. Thus, in our study, the difference in hydrophobicity/hydrophilicity character of PVDF scaffolds and coverslip can explain the different cell behavior against the two types of biomaterials.

#### 3.4.2. Cell Adhesion and Morphology

**Figure 7:**
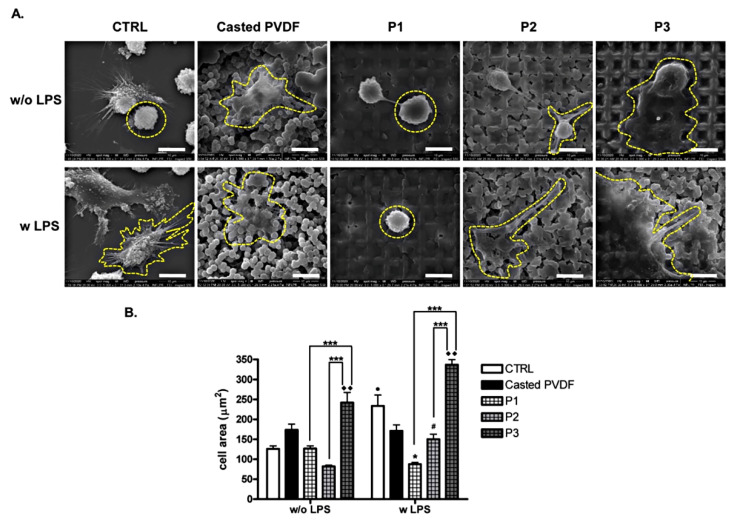
(**A**) Representative images of THP-1 derived macrophage cells morphology on surface materials seen by SEM microscopy at 5000× magnification (Inspect S Electron Scanning Microscope). Scale bar is 10 μm.; (**B**). Quantification of cell area (μm^2^) • *p* < 0.05 vs. CTRL w/o LPS, * *p* < 0.05 vs. P1 w/o LPS, # *p* < 0.05 vs. P2 w/o LPS, ♦♦ *p* < 0.01 vs. P3 w/o LPS, *** *p* < 0.001 P1 vs. P3 and P2 vs. P3 in inflammatory and non-inflammatory conditions.

Substrate properties and topography in particular influence cell adhesion, shape, and activity with cell type specificity. Biomaterial’s surface combined parameters can affect macrophages morphology and phenotype, and thus inducing a change in cell response to biomaterial [18,48]. Here we examined the effect of surface topography on the adhesion and morphology of THP-1 cells attachment by SEM in normal and inflammatory conditions, respectively. After 2 days of THP-1 cells differentiation in direct contact with the surface followed by incubation with medium or LPS for 18 h, macrophages were subjected to scanning electron microscopy. As shown in Figure 7A in the absence of inflammatory stimulus, cells grown on P1, P2, and CTRL showed a small round morphology that covered one topographic element with an area of ~100 μm^2^ (Figure 7B). In the case of P3, cells developed a larger area, flatted morphology which covered several topographical elements (~250 μm^2^), a statistical increase (*** *p* < 0.001) compared to P1 and P2. In the case of LPS stimulation, as topographic depth increases, the cell area increases (*** *p* < 0.001 P1 vs. P3 and P2 vs. P3). It can be observed that cells exhibit distinctive cell morphologies for different samples. Thus, macrophages grown on control and casted polymer surfaces showed a more polarized morphology with discrete cytoplasmatic extensions that might be associated with a pro-inflammatory phenotype with an area of up to 250 μm^2^. Contrary to that, on P1 sample, cells presented predominantly spherical morphology similar to that found for unstimulated cells. In the case of P2 surfaces, macrophages have a tendency to spread, acquiring long stretching filopodia (~150 μm^2^). More extended morphology with spread cells with protruding filopodia were found on P3 samples in inflammatory simulated condition with the area over 300 μm^2^. In conclusion, statistical relevant differences in cell area were found for topographically modified surfaces between non-inflammatory and inflammatory conditions (Figure 7B).

It has been reported that topography design and size scale profile induce specific morphological cellular responses that can modulate macrophage activity [49,50,51,52]. Distinctive morphologies of primary human macrophages were observed on nano- and micro-textured PVDF surface with a correlation with inflammatory activity [16]. Malheiro et al. [18] showed that concave and convex structures controlled human THP-1 macrophages shape but did not affect cell phenotype. Instead, THP-1 cells morphology changed on micro and nano-patterned titanium surfaces [53], with cell polarization [51,53]. Different scales of a honeycomb pattern influenced macrophage cell spreading and resulted in distinctive cell morphologies associated with polarization state [54]. Previously Luu et al. showed that topography cues of the surface altered macrophage cell morphology and polarization state [48].

#### 3.4.3. Cytokine Release

**Figure 8:**
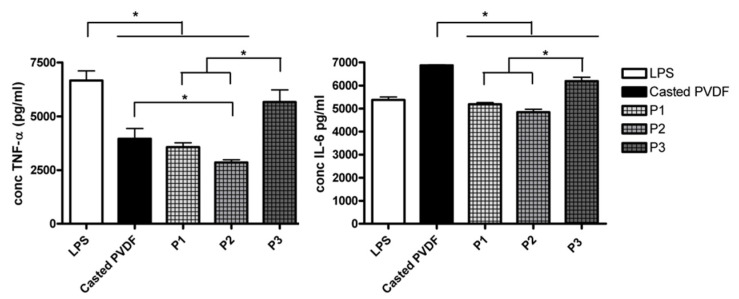
Secreted levels of pro-inflammatory cytokines TNF-α and IL-6 from THP-1 cell cultured on surface materials in lipopolysaccharide (LPS) treated condition. Data are presented as mean values ± SD, and significance was determined at * *p* < 0.05.

Further, we have investigated if the surface topography of PVDF coating modulates the macrophage pro-inflammatory cytokine release in vitro. To assess cell reaction to material surfaces, macrophages were grown on PVDF coatings and stimulated with LPS (10 ng/mL). The level of pro-inflammatory cytokines TNF- α and IL-6 in cell supernatants was measured after 18 h of incubation. Results depicted in Figure 8 revealed the influence of topography on macrophages immune response. Thus, TNF-α level was lower for all the PVDF scaffolds compared to the control (cover slip) *p* < 0.05, the lowest level being obtained for P2 sample. In the case of IL-6, the response was similar to those obtained for the control, excepting the casted polymer. Among the topographically modified coatings, P1 and P2 induced a significant reduction of IL-6 level compared to the casted or P3 (*p* < 0.05). In the absence of LPS treatment, no detectable levels of cytokines released by THP-1 cells regardless of type of surfaces were recorded (Supplementary data, Table S1).

It is very well known that the characteristics of biomaterial such as topography, internal architecture, surface charge, and chemical composition are key parameters that influence the activation status of macrophages [34]. By designing materials with appropriate surface properties, the behavior of macrophages may be modulated to promote a specific, desired immune response to biomaterial implants [55]. Thus, it has been reported that biomaterials with hydrophilic surfaces induce less inflammation while the hydrophobic surfaces are associated with the up-regulation of the pro-inflammatory response by macrophages. Roughness and porosity of biomaterial surfaces also modulate the inflammatory response of macrophages [26]. By manipulating these parameters, biomaterials with enhanced biological activity can be designed [39]. In our study the level of both TNF-α and IL-6 cytokines was decreased in the case of P2 scaffold and increased for P3 sample, which exhibits the highest roughness (1 µm) and inverted pyramidal depth (2.5µm) among the modified surfaces, characteristics which resulted in driving macrophages toward a pro-inflammatory phenotype. The observed difference in cytokines secretion may be also the result differences in cell morphology, macrophages attached on P3 exhibiting well spreading, extended cell body, and increased filopodia extension (Figure 7). These findings are in line with the observations that enhanced macrophages activation toward a pro-inflammatory state is favored by surface roughness associated with LPS stimulation [56], pore size, and hydrophobicity [57,58] macrophages shape and spreading induced by topography [39,59].

## 4. Conclusions

In this work, the controlled fabrication of polyvinylidene fluoride (PVDF) nano-microstructured biointerfaces obtained by replication method was described, highlighting how the physical-chemical characteristics of porous replicated PVDF biointerfaces can induce the change in hydrophobicity and in vitro immunomodulation of macrophages. Three types of squared wells like structures with depths of 0.8 to 2.5 microns were obtained, displaying not only the micron features of the mold but also globular and porous internal nanoarchitecture. No significant chemical modifications were observed for the replicated substrates, but a decrease of the contact angle with 30% for the replicated substrates with 2.5 microns depth.

Biological investigations revealed that cell viability, morphology, and immune response are modulated by topographical characteristics of the material surfaces. Among the topographically modified PVDF scaffolds, the sample with the depth of 1.5 microns and a moderate hydrophobic surface was found to induce the lowest pro-inflammatory cell response. These results bring the perspective of tailoring the topographical surface properties in order to achieve a synergetic immune and adequate future bone formation response.

Collectively, our results highlight the potential of the porous bio-structured PVDF materials as valuable candidates for bone tissue engineering and other medical applications.

## Figures and Tables

**Table 1:** Summary of Replica PVDF β, γ- phases.

β-Phase	Replicated PVDF
839 cm^−1^	related to C–C–C asymmetrical stretching vibration and CF stretching vibration (deformation of CF_2_)
1071 and 1178 cm^−1^	symmetrical stretching of CF_2_ group
**γ-Phase**	**Replicated PVDF**
1233 cm^−1^	γ- polymorph phase

**Table 2:** C/F and O/C ratio in the pristine and replicated PVDF.

PVDF	Atomic Ratio	
	F/C	O/C
Pristine	0.86	0.08
Replicated	1.36	0.46

## Data Availability

Data is contained within the article or Supplementary Materials.

## Supplementary Materials

The following are available online at https://www.mdpi.com/article/10.3390/nano11081913/s1, Figure S1: SEM micrographs images (right) SYLGARD™ 184 Silicone Elastomer mold shaped as pyramidal microarrays having a lateral size of 8 microns. Table S1. Summary of mean values and standard deviation (SD) values of secreted levels of pro-inflammatory cytokines TNF-α and IL-6 from THP-1 cell cultured on surface materials in lipopolysaccharide treated (w LPS) and non-treated condition (w/o LPS).
