# Supplementary material for: In Vitro Effect of Replicated Porous Polymeric Nano-MicroStructured Biointerfaces Characteristics on Macrophages Behavior
Source: Nanomaterials (Basel). 2021 Jul 25;11(8):1913. doi: 10.3390/nano11081913 (PMC8400858; doi:10.3390/nano11081913)
Supplement: Supplementary file 1 [file nanomaterials-11-01913-s001.zip › supplementary.pdf]

Supplementary Materials

In Vitro Effect of Replicated Porous Polymeric Nano-Micro-Structured Biointerfaces Characteristics on Macrophages Behavior

Luminita Nicoleta Dumitrescu [1], Madalina Icriverzi [2], Anca Bonciu [1,3,4], Anca Roșeanu [2,*], Antoniu Moldovan [1] and Valentina Dinca [1,3,*]

[1] National Institute for Lasers, Plasma, and Radiation Physics, 409 Atomiştilor Street, 077125 Magurele, Romania; nicoleta.dumitrescu@inflpr.ro (L.N.D.); anca.bonciu@inflpr.ro (A.B.); antoniu.moldovan@inflpr.ro (A.M.)

[2] Institute of Biochemistry of the Romanian Academy of Sciences, 060031 Bucharest, Romania; radu_mada@yahoo.co.uk

[3] FOTOPLASMAT Center, 409 Atomiştilor Street, 077125 Magurele, Romania

[4] Faculty of Physics, University of Bucharest, 405 Atomistilor, 077125 Magurele, Romania

* Correspondence: roseanua@gmail.com (A.R.); valentina.dinca@inflpr.ro (V.D.); Tel.: +402-1457-4414 (V.D.)

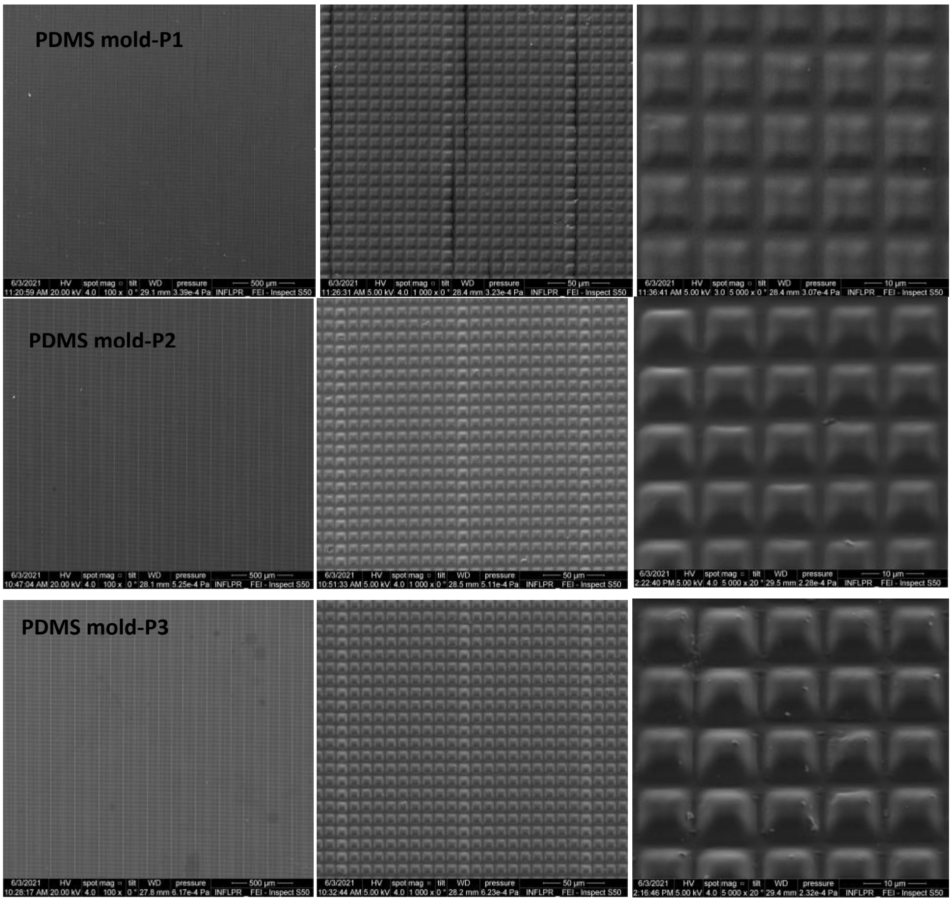

Figure S1. SEM micrographs images (right) SYLGARD™ 184 Silicone Elastomer mold shaped as pyramidal microarrays having a lateral size of 8 microns.

Table S1. Summary of mean values and standard deviation (SD) values of secreted levels of pro-inflammatory cytokines TNF-α and IL-6 from THP-1 cell cultured on surface materials in lipopolysaccharide treated (w LPS) and non-treated condition (w/o LPS).

TNF-α pg/ml	Mean values ± SD (pg/mL)	
	w/o LPS	w LPS

CTRL	−3.68 ± 0.54	6659.73 ± 450.10
Casted PVDF	−2.81 ± 0.41	3950.76 ± 670.67
P1	−2.71 ± 0.22	3568.33 ± 344.91
P2	−3.68 ± 0.08	2854.17 ± 214.46
P3	−3.87 ± 0.41	5665.60 ± 975.99
**IL-6 pg/ml**	**Mean values ± SD (pg/mL)**	
	**w/o LPS**	**w LPS**
CTRL	−6.69 ± 0.20	5374.33 ± 130.01
Casted PVDF	−5.65 ± 0.38	6881.70 ± 22.23
P1	−6.09 ± 0.41	5188.48 ± 71.85
P2	−6.05 ± 0.48	4845.16 ± 169.87
P3	−5.53 ± 0.42	6190.96 ± 241.12
